# Evaluating Primary Treatment for People with Advanced Glaucoma: Five-Year Results of the Treatment of Advanced Glaucoma Study

**DOI:** 10.1016/j.ophtha.2024.01.007

**Published:** 2024-07

**Authors:** Anthony J. King, Jemma Hudson, Augusto Azuara-Blanco, Jennifer Burr, Ashleigh Kernohan, Tara Homer, Hosein Shabaninejad, John M. Sparrow, David Garway-Heath, Keith Barton, John Norrie, Tracey Davidson, Luke Vale, Graeme MacLennan, TAGS Study Group∗

**Affiliations:** 1 Nottingham University Hospital, Nottingham, United Kingdom; 2 Centre for Healthcare Randomised Trials (CHaRT), Health Services Research Unit, University of Aberdeen, Aberdeen, United Kingdom; 3 Centre for Public Health, Queen’s University Belfast, Royal Victoria Hospital, Belfast, United Kingdom; 4 School of Medicine, University of St. Andrews, St. Andrews, United Kingdom; 5 Health Economics Group, Population Health Sciences Institute, Newcastle University, Newcastle upon Tyne, United Kingdom; 6 Bristol Eye Hospital, University Hospitals Bristol NHS Foundation Trust, Bristol, United Kingdom; 7 National Institute for Health Research (NIHR) Biomedical Research Centre, Moorfields Eye Hospital NHS Foundation Trust and UCL Institute of Ophthalmology, London, United Kingdom; 8 Edinburgh Clinical Trials Unit, Usher Institute, University of Edinburgh, Edinburgh, United Kingdom

**Keywords:** Intraocular pressure, Open-angle glaucoma, Quality of life, Randomized controlled trial, Visual field loss

## Abstract

**Purpose:**

To determine whether primary trabeculectomy or medical treatment produces better outcomes in terms of quality of life (QoL), clinical effectiveness, and safety in patients with advanced glaucoma.

**Design:**

Multicenter randomized controlled trial.

**Participants:**

Between June 3, 2014, and May 31, 2017, 453 adults with newly diagnosed advanced open-angle glaucoma in at least 1 eye (Hodapp classification) were recruited from 27 secondary care glaucoma departments in the United Kingdom. Two hundred twenty-seven were allocated to trabeculectomy, and 226 were allocated medical management.

**Methods:**

Participants were randomized on a 1:1 basis to have either mitomycin C-augmented trabeculectomy or escalating medical management with intraocular pressure (IOP)-reducing drops as the primary intervention and were followed up for 5 years.

**Main Outcome Measures:**

The primary outcome was vision-specific QoL measured with the 25-item Visual Function Questionnaire (VFQ-25) at 5 years. Secondary outcomes were general health status, glaucoma-related QoL, clinical effectiveness (IOP, visual field, and visual acuity), and safety.

**Results:**

At 5 years, the mean ± standard deviation VFQ-25 scores in the trabeculectomy and medication arms were 83.3 ± 15.5 and 81.3 ± 17.5, respectively, and the mean difference was 1.01 (95% confidence interval [CI], –1.99 to 4.00; *P* = 0.51). The mean IOPs were 12.07 ± 5.18 mmHg and 14.76 ± 4.14 mmHg, respectively, and the mean difference was –2.56 (95% CI, –3.80 to –1.32; *P* < 0.001). Glaucoma severity measured with visual field mean deviation were –14.30 ± 7.14 dB and –16.74 ± 6.78 dB, respectively, with a mean difference of 1.87 (95% CI, 0.87–2.87 dB; *P* < 0.001). Safety events occurred in 115 (52.2%) of patients in the trabeculectomy arm and 124 (57.9%) of patients in the medication arm (relative risk, 0.92; 95% CI, 0.72–1.19; *P* = 0.54). Serious adverse events were rare.

**Conclusions:**

At 5 years, the Treatment of Advanced Glaucoma Study demonstrated that primary trabeculectomy surgery is more effective in lowering IOP and preventing disease progression than primary medical treatment in patients with advanced disease and has a similar safety profile.

**Financial Disclosure(s):**

Proprietary or commercial disclosure may be found in the Footnotes and Disclosures at the end of this article.

Sight loss resulting from glaucoma is often preventable with early diagnosis and treatment. However, because glaucoma is asymptomatic in its early phases, people often are unaware of its onset, leading to presentation with more advanced disease. In the United Kingdom, around 25% of patients with glaucoma demonstrate advanced disease in at least 1 eye at presentation.^1^^,^^2^ Presentation with advanced glaucoma is associated with socioeconomic deprivation^3^ and is the main risk factor for progression to blindness.^4^

Reducing intraocular pressure (IOP) is the only proven effective treatment for glaucoma. Better IOP control at an early stage reduces the risk of further progression.^5^ Primary treatment options for presentation with advanced glaucoma mainly are medical or surgical interventions. The Preferred Practice Patterns of the American Academy of Ophthalmology^6^ do not recommend a specific treatment approach for those who present with advanced disease, whereas the European Glaucoma Society guidelines^7^ suggests that trabeculectomy can be considered in patients presenting with advanced glaucoma. In the United Kingdom, the National Institute for Health and Care Excellence guidelines suggest patients presenting with advanced disease should consider trabeculectomy as a primary intervention^8^ but cite poor evidence to support this recommendation. Most United Kingdom ophthalmologists do not follow this guidance and choose to treat patients medically with escalating topical medication therapy,^9^ only offering trabeculectomy if medical management is not successful. This approach is the result of the poor evidence base supporting trabeculectomy as a primary intervention and concern regarding surgical complications. However, clinicians indicated that high-quality evidence would change their practice.^9^

In a Cochrane systematic review^10^ comparing primary medical versus surgical treatment for open-angle glaucoma (OAG), the authors concluded that trabeculectomy lowers IOP more than medication but also that trials excluded patients with advanced disease and did not reflect current medical and surgical practice. Comparison of current medical options and modern trabeculectomy in people with advanced OAG was identified as a research priority.^10^

We carried out a multicenter randomized controlled trial to compare primary medical management against primary trabeculectomy for people with advanced OAG, evaluating patient reported outcomes, clinical effectiveness, and safety. Patients recruited to the Treatment of Advanced Glaucoma Study (TAGS) on average were 67 years of age at diagnosis and were representative of the eligible patient population.^11^ A previous report at 24 months showed a lower IOP in the trabeculectomy arm, but no evidence of a difference in disease progression or in any other clinical or QoL measurement.^12^^,^^13^ In this article, we compare long-term outcomes.

## Methods

### Study Design and Participants

The TAGS is a multicenter, parallel-group, open-label, pragmatic randomized controlled trial in 27 hospitals in the United Kingdom. The study was approved by the East Midlands Derby Research Ethics Committee (reference no., 13/EM/0395). The study was conducted in accordance with good clinical practice guidelines and adhered to the tenets of the Declaration of Helsinki. All patients provided written informed consent before participation. Two independent committees oversaw the trial. An independent data and safety monitoring committee appraised adverse events (AEs) and reported to an independent trial steering committee. This study was registered with the International Standard Randomised Controlled Trial Number (ISRCTN) registry (identifier, ISRCTN56878850). The protocol^14^ was published previously, and the methods are summarized below.

### Participants

Participants were adults with severe glaucoma according to the extent of visual field loss (Hodapp-Parrish-Anderson classification)^15^ in 1 or both eyes at presentation. Inclusion criteria included having a diagnosis of OAG (including pigment dispersion glaucoma, pseudoexfoliative glaucoma, and normal-tension glaucoma), being willing to participate in a trial, being able to provide informed consent, and being 18 years or older. Patients were excluded if they were unable to undergo incisional surgery; had a high risk of trabeculectomy failure such as previous conjunctival surgery or complicated cataract surgery; had secondary glaucoma or primary angle-closure glaucoma; or were pregnant, nursing, or planning a pregnancy or were women and of childbearing potential not using a reliable method of contraception.

### Advanced Glaucoma

Severe glaucomatous visual field loss (Hodapp-Parrish-Anderson classification)^15^ was defined according to the following criteria: mean deviation (MD) of < −12.00 dB, > 50% of points defective in the pattern deviation probability plot at the 5% level (> 27 points on 24-2 Humphrey Visual Field [HVF] analyzer, Carl Zeiss Meditec), > 20 points defective at the 1% level; a point in the central 5° with a sensitivity of 0 dB; and points within 5° of fixation of < 15-dB sensitivity in both upper and lower hemifields.

### Randomization and Masking

Participants were assigned randomly (1:1) to trabeculectomy or medical management with a minimization algorithm based on center and presence of bilateral disease. The unit of randomization was the participant (not the eye). For participants with both eyes eligible, an index eye was selected based on less severe disease according to the MD value of the visual field (VF), but both eyes would receive the same allocated treatment. For those in the trabeculectomy arm of the study, it was planned that the index eye underwent surgery first.

Participants were enrolled by trained center staff (local principal investigator, research nurse, or proxy) who used a remote web-based application, or an interactive voice response telephone system located at the Centre for Healthcare Randomised Trials (University of Aberdeen, Aberdeen, United Kingdom) for group allocation.

Intraocular pressure measurement was undertaken with Goldman tonometry and was masked to the intervention according to the 2-observer technique^14^ to avoid bias. Visual field assessment was undertaken using the HVF analyzer 24-2 SITA standard algorithm, and evaluation of VF progression was undertaken by an independent reading center that was not aware of allocation assignment of participants (Central Administrative Research Facility, Queens University, Belfast, United Kingdom). The reading center assessed progression on the basis of HVF MD change. Surgeons and participants could not be masked to the allocated procedure because of the nature of the interventions.

### Procedures

After the diagnosis of advanced glaucoma was made, potential participants were administered holding medical treatment. After randomization, participants allocated to trabeculectomy were added to the National Health Service surgical waiting list and continued holding medication to lower IOP until trabeculectomy was undertaken. We anticipated that surgery would occur within 3 months of randomization. Each operating surgeon was a fellowship-trained glaucoma specialist experienced in undertaking standard trabeculectomy. A surgical technique questionnaire was completed by all potential surgeons to ensure that recognized standard trabeculectomy procedures^16^ were followed. These questionnaires were reviewed and signed off by the chief investigator; no feedback was given because all surgeons essentially were conducting the same operation. The technique used was not modified for the purposes of the trial. All other aspects of care were left to the discretion of the responsible surgeon.

The definition of standard trabeculectomy included the fashioning of a guarded fistula and augmentation with mitomycin C. The exposure time and concentration of mitomycin C were left to the discretion of the operating surgeon. A small hole into the anterior chamber was created that was covered by a flap of partial thickness sclera, allowing aqueous humor to filter into the subconjunctival space. The operation could be performed under either local or general anaesthetic. For participants with bilateral advanced glaucoma allocated to the trabeculectomy arm, it was expected that the index eye would undergo surgery first; however, the final decision was made by the treating surgeon, in discussion with the patient, about which eye would undergo trabeculectomy first.

Participants randomized to the medical treatment arm underwent an escalating medical management regimen and were prescribed a variety of topical glaucoma medications in accordance with accepted standard of care according to National Institute for Health and Care Excellence guidelines,^8^ European Glaucoma Society guidelines,^7^ and Preferred Practice Patterns of the American Academy of Ophthalmology.^6^ Escalation of medical management was based on the judgement of the treating clinician. When topical medications failed to control IOP adequately, oral carbonic anhydrase inhibitors could be used. If IOP control was deemed inadequate with maximum medical therapy, trabeculectomy was offered. IOP targets were guided by the “Canadian Perspectives in Glaucoma Management: Setting Target IOP Range” consensus.^17^

### Outcomes

The primary outcome was vision-related quality of life (QoL) measured with the 25-item Visual Function Questionnaire (VFQ-25).^18^ The secondary outcomes included other patient-reported outcomes measured with the EQ-5D-5L,^19^ the Health Utility Index Mark 3,^20^ the Glaucoma Utility Index,^21^ the VFQ-25, and patient experience. The VFQ-25 was measured at baseline and 4, 12, 24, 36, 48, and 60 months after randomization, whereas the remainder were measured at baseline and 1, 3, 6, 12, 18, 24, 36, 48, and 60 months after randomization.

Patient-reported outcomes were assessed with self-completed questionnaires at baseline (before randomization) and at follow-up clinic visits. Additionally, postal questionnaires were sent to participants at 1, 3, 6, 18, 36, and 48 months after randomization.

The clinical effectiveness outcomes were IOP, logarithm of the minimum angle of resolution visual acuity, glaucoma severity according to VF MD measured with the HVF analyzer, need for cataract surgery, accordance with visual standards for driving (based on Esterman VF), eligibility for sight impairment certification,^22^ and safety of interventions. These were measured at baseline and 4, 12, 24, and 60 months. Adverse events were recorded by the local research team and through follow-up questionnaires completed by the participants. Events related to participating in the trial or related to glaucoma were considered to be AEs.

The local research team at each center collected data at baseline and at scheduled follow-up visits at 4, 12, 24, and 60 months after randomization. Data collected at follow-up visits included postoperative interventions, related hospital readmissions, and medication changes.

### Statistical Analysis

The sample size was constrained by the initial 2-year TAGS follow-up: we planned to randomize 440 patients, allowing for a 13.5% attrition rate, to obtain 90% power for a 2-sided 5% significance level to detect a 6-point difference on the VFQ-25, assuming a common standard deviation (SD) of 18 points.^23^ We summarized outcome data using mean ± SD for continuous data, frequencies and percentages for categorical variables, and line plots to visualize outcomes over time. Outcomes were analyzed with a heteroscedastic partially nested repeated measures mixed-effects linear model,^24^ correcting for the baseline measure of the outcome and bilateral disease severity and including a random effect for surgeon by using restricted maximum likelihood. For visual standards for driving and need for drops at 60 months, we used a Poisson model adjusting for bilateral disease and including a random effect for surgeon to estimate relative risk. For amount of cataract surgery, safety, and further surgery, because participants had varying follow-up times, data were presented as number (probability) and were analyzed using Cox regression adjusting for bilateral disease and presented as risk ratios.^25^ For certification as sight impaired, we used the Fisher exact test to compare groups. All estimates were presented with 95% confidence intervals (CIs). Subgroup analysis for the primary outcome is reported for variables shown in Figure S1 (available at www.aaojournal.org), using a stricter level of statistical significance (2-sided 1% significance level) and 99% CIs. Sensitivity analysis of the primary outcome (VFQ-25) and clinical outcomes (IOP and VF) explored missing data using multiple imputation using chained equations. We used Stata version 17 software (StataCorp LLC) for all analyses.

## Results

**Figure 2:**
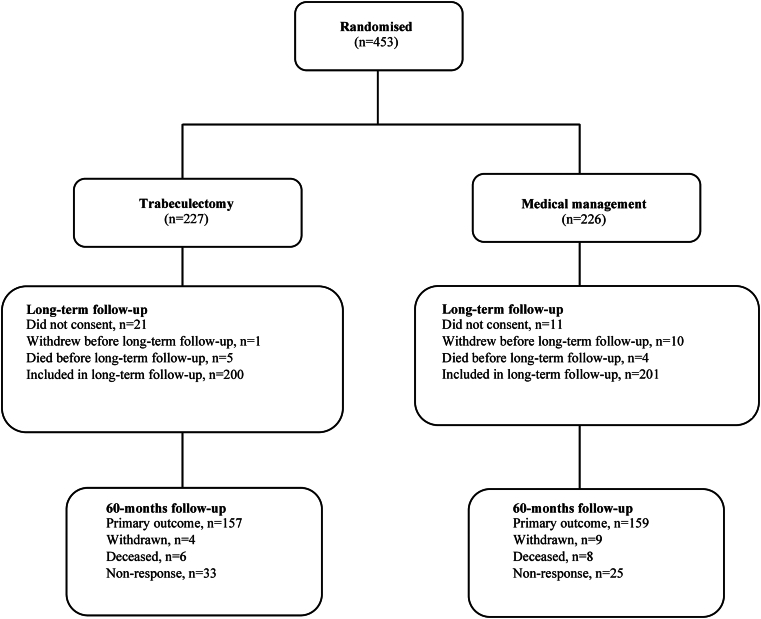
Consolidated Standards of Reporting Trials diagram for the Treatment of Advanced Glaucoma Study.

Between June 3, 2014, and May 31, 2017, 453 participants from 27 hospitals were allocated to either trabeculectomy (n = 227) or medical management (n = 226; Fig 2). In the trabeculectomy arm, 201 participants (88.5%) underwent trabeculectomy on the index eye. All participants received the allocated treatment in the medical management arm. In total, 401 participants (88.5%) agreed to extended follow-up beyond 2 years. At baseline, both arms were evenly matched for all clinical, demographic, and QoL variables.^12^ Forty-four participants (19.4%) were in the trabeculectomy arm, and 40 participants (19.5%) were in the medical management arm who had advanced glaucoma in both eyes.

At 5 years, the mean ± SD VFQ-25 was 83.3 ± 15.5 and 81.3 ± 17.5 in the trabeculectomy and the medical management arms, respectively. The groups’ mean difference was 1.01 (95% CI, –1.99 to 4.00; *P* = 0.51; Table 1). Table S2 (available at www.aaojournal.org) shows the results for VFQ-25 subscales at 5 years, which did not reveal treatment effect differences apart from the driving subscale, which suggested that those in the medical arm were less likely to be driving at 5 years (trabeculectomy and medical arms, 78.3 ± 29.8 and 67.9 ± 37.2, respectively; mean difference, 8.93; *P* < 0.04).

**Table 1:** Quality-of-Life Outcomes

Outcome	Trabeculectomy (n = 227)		Medical Management (n = 226)		ES∗	95% Confidence Interval	*P* Value
	Mean ± Standard Deviation	No.	Mean ± Standard Deviation	No.			
VFQ-25†							
Baseline	87.1 ± 13.6	226	87.1 ± 13.4	224			
4 mos	85.1 ± 14.9	212	86.5 ± 13.6	216	–1.13	–3.85 to 1.59	0.42
12 mos	85.4 ± 14.3	214	86.3 ± 13.1	209	–0.49	–3.23 to 2.24	0.72
24 mos	85.4 ± 13.8	207	84.5 ± 16.3	205	1.15	–1.60 to 3.91	0.41
36 mos	84.1 ± 15.8	159	83.6 ± 16.2	152	–0.01	–3.03 to 3.00	0.99
48 mos	84.6 ± 14.7	138	81.9 ± 17.0	142	1.94	–1.17 to 5.04	0.22
60 mos	83.3 ± 15.5	157	81.3 ± 17.5	159	1.01	–1.99 to 4.00	0.51
EQ-5D-5L							
Baseline	0.844 ± 0.185	222	0.837 ± 0.176	222			
1 mos	0.838 ± 0.185	194	0.808 ± 0.203	203	0.024	–0.013 to 0.062	0.21
3 mos	0.836 ± 0.167	186	0.814 ± 0.195	179	0.014	–0.024 to 0.053	0.47
6 mos	0.850 ± 0.184186	186	0.822 ± 0.204	195	0.016	–0.022 to 0.055	0.40
12 mos	0.837 ± 0.177	211	0.823 ± 0.164	209	0.014	–0.023 to 0.051	0.45
18 mos	0.828 ± 0.185	181	0.791 ± 0.219	184	0.022	–0.017 to 0.061	0.26
24 mos	0.810 ± 0.179	206	0.796 ± 0.191	203	0.015	–0.022 to 0.053	0.43
36 mos	0.806 ± 0.186	156	0.787 ± 0.207	151	0.017	–0.024 to 0.058	0.43
48 mos	0.820 ± 0.160	135	0.769 ± 0.192	140	0.042	–0.001 to 0.084	0.05
60 mos	0.787 ± 0.191	151	0.765 ± 0.187	152	0.016	–0.025 to 0.057	0.44
HUI-3							
Baseline	0.814 ± 0.202	214	0.809 ± 0.208	214			
1 mos	0.791 ± 0.232	184	0.786 ± 0.230	193	–0.003	–0.048 to 0.042	0.89
3 mos	0.796 ± 0.223	180	0.779 ± 0.222	179	0.004	–0.042 to 0.050	0.87
6 mos	0.805 ± 0.216180	180	0.782 ± 0.224	182	0.017	–0.029 to 0.063	0.47
12 mos	0.829 ± 0.193	204	0.798 ± 0.199	196	0.021	–0.023 to 0.066	0.35
18 mos	0.802 ± 0.212	169	0.749 ± 0.258	174	0.019	–0.028 to 0.066	0.42
24 mos	0.786 ± 0.227	198	0.751 ± 0.246	193	0.033	–0.012 to 0.077	0.15
36 mos	0.769 ± 0.229	154	0.747 ± 0.220	145	0.011	–0.038 to 0.060	0.66
48 mos	0.756 ± 0.239	132	0.709 ± 0.247	138	0.026	–0.024 to 0.076	0.31
60 mos	0.726 ± 0.262	150	0.706 ± 0.239	147	0.001	–0.048 to 0.050	0.97
GUI							
Baseline	0.897 ± 0.127	219	0.886 ± 0.120	222			
1 mos	0.876 ± 0.142	194	0.870 ± 0.151	205	0.004	–0.025 to 0.032	0.81
3 mos	0.864 ± 0.129	187	0.861 ± 0.152	190	–0.003	–0.032 to 0.026	0.82
6 mos	0.856 ± 0.154	186	0.868 ± 0.130	191	–0.015	–0.044 to 0.014	0.30
12 mos	0.875 ± 0.132	209	0.875 ± 0.135	204	–0.002	–0.030 to 0.026	0.88
18 mos	0.866 ± 0.142	181	0.853 ± 0.148	184	0.003	–0.026 to 0.033	0.82
24 mos	0.861 ± 0.151	205	0.845 ± 0.175	202	0.016	–0.012 to 0.044	0.26
36 mos	0.849 ± 0.143	158	0.827 ± 0.162	150	0.008	–0.023 to 0.039	0.61
48 mos	0.855 ± 0.141	135	0.828 ± 0.167	139	0.018	–0.014 to 0.050	0.28
60 mos	0.858 ± 0.127	155	0.826 ± 0.161	151	0.023	–0.009 to 0.054	0.16
Patient experience (glaucoma worsening)	No./Total No. (%)		No./Total No. (%)				
Baseline	95/208 (45.7)		76/209 (36.4)				
1 mos	60/188 (31.9)		50/201 (24.9)		1.12	0.73–1.71	0.60
3 mos	37/182 (20.3)		40/185 (21.6)		0.82	0.51–1.34	0.44
6 mos	30/182 (16.5)		40/189 (21.2)		0.69	0.41–1.16	0.16
12 mos	38/207 (18.4)		57/199 (28.6)		0.55	0.35–0.87	0.01
18 mos	40/180 (22.2)		38/181 (21.0)		0.94	0.58–1.53	0.80
24 mos	44/196 (22.4)		57/194 (29.4)		0.66	0.42–1.02	0.06
36 mos	39/153 (25.5)		43/143 (30.1)		0.77	0.48–1.24	0.29
48 mos	42/135 (31.1)		45/140 (32.1)		0.86	0.54–1.37	0.53
60 mos	39/147 (26.5)		53/145 (36.6)		0.64	0.40–1.01	0.06

ES = effect size; EQ-5D-5L = EuroQoL-5 Dimension-5 Level; GUI = Glaucoma Utility Index; HUI-3 = Health Utility Index Mark 3; VFQ-25 = 25-item Visual Function Questionnaire.

∗ Mean difference for continuous outcomes and risk ratios for dichotomous outcomes.

† Primary outcome.

Also, no evidence was found of any differences between the prespecified subgroups (Fig S1, available at www.aaojournal.org). Sensitivity analysis of multiple imputation showed similar results (Table S3, available at www.aaojournal.org).

**Figure 3:**
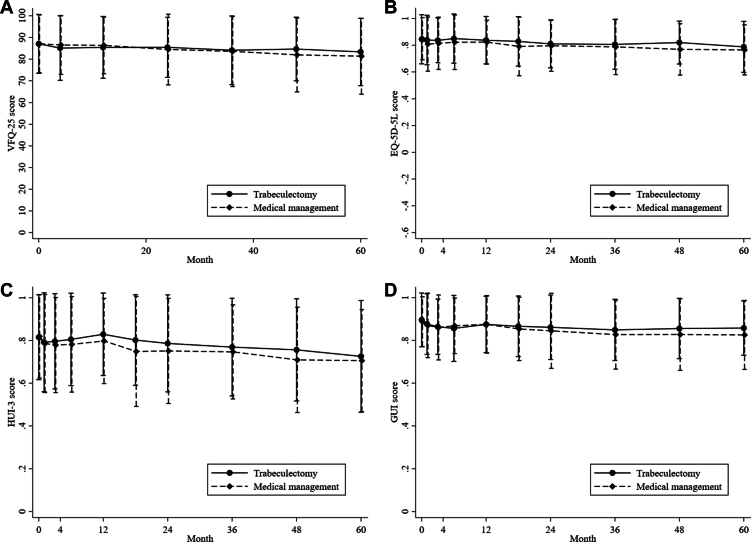
**A**–**D**, Graphs showing quality-of-life outcomes up to 5 years for (**A**) the 25-item Visual Function Questionnaire (VFQ-25), (**B**) the EQ-5D-5L, (**C**) Health Utility Index Mark 3 (HUI-3), and (**D**) the Glaucoma Utility Index (GUI).

For the EQ-5D-5L at 5 years, the mean ± SD score was 0.787 ± 0.191 and 0.765 ± 0.187 in the trabeculectomy and medical management arms, respectively; the mean difference was 0.016 (95% CI, –0.025 to 0.057; *P* = 0.44; Table 1; Fig 3). Similarly, for the Health Utility Index Mark 3 and Glaucoma Utility Index, the mean scores were higher in the trabeculectomy arm, but the differences were not statistically significant (Table 1; Fig 3). A reduction was found in the number of participants in the trabeculectomy arm who thought the glaucoma was become worse at 5 years compared with that of the baseline but not in the medical management arm (relative risk [RR], 0.64; 95% CI, 0.40–1.01; *P* = 0.06).

The mean ± SD IOP at 5 years was 12.07 ± 5.18 mmHg for the trabeculectomy arm and 14.76 ± 4.14 mmHg for the medical management arm; the mean difference was –2.56 (95% CI, –3.80 to –1.32; *P* < 0.001; Table 4; Fig 3). Sensitivity analyses of multiple imputations were consistent with the main analysis (Table S3).

**Table 4:** Clinical Outcomes

Outcome	Trabeculectomy (n = 227)		Medical Management (n = 226)		ES∗	95% Confidence Interval	*P* Value
	Mean ± Standard Deviation	No.	Mean ± Standard Deviation	No.			
Intraocular pressure (mmHg)							
Diagnosis†	26.9 ± 9.1	226	25.9 ± 8.4	223			
Baseline	19.40 ± 6.15	222	19.05 ± 5.73	221			
4 mos	12.39 ± 5.73	217	16.40 ± 4.12	220	–4.04	–5.17 to –2.91	< 0.001
12 mos	11.94 ± 4.48	215	16.12 ± 4.54	209	–4.19	–5.33 to –3.05	< 0.001
24 mos	12.40 ± 4.71	206	15.07 ± 4.80	202	–2.67	–3.82 to –1.51	< 0.001
60 mos	12.07 ± 5.18	166	14.76 ± 4.14	162	–2.56	–3.80 to –1.32	< 0.001
Visual acuity (logMAR)							
Baseline	0.15 ± 0.25	227	0.17 ± 0.26	223			
4 mos	0.25 ± 0.31	210	0.16 ± 0.24	217	0.09	0.04 to 0.14	< 0.001
12 mos	0.18 ± 0.23	212	0.16 ± 0.26	209	0.03	–0.02 to 0.08	0.26
24 mos	0.21 ± 0.28	199	0.16 ± 0.26	201	0.06	0.01 to 0.12	0.014
60 mos	0.19 ± 0.23	138	0.20 ± 0.32	136	0.02	–0.04 to 0.08	0.48
Visual fields mean deviation (dB)							
Baseline	–14.91 ± 6.36	227	–15.26 ± 6.34	226			
4 mos	–14.35 ± 6.78	211	–14.84 ± 6.52	217	0.05	–0.85 to 0.95	0.92
12 mos	–14.76 ± 6.92	214	–14.95 ± 6.53	209	0.13	–0.77 to 1.03	0.77
24 mos	–15.15 ± 6.63	202	–15.42 ± 6.39	200	0.29	–0.63 to 1.20	0.54
60 mos	–14.30 ± 7.14	144	–16.74 ± 6.78	145	1.87	0.87 to 2.87	< 0.001
	No./Total No. (%)		No./Total No. (%)				

Need for cataract surgery							
Yes	57 (26.9)		56 (27.5)		0.99	0.69–1.43	0.97
Visual standards for driving at 60 mos							
Yes	161/178 (90.4)		162/182 (89.0)		1.02	0.82–1.27	0.84
Certified as SI at 60 mos							
No	168/175 (96.0)		171/177 (96.6)				0.91
SI	5/175 (2.9)		5/177 (2.8)				
Severe SI	2/175 (1.1)		1/177 (0.6)				
Eligible to be certified as SI at 60 mos							
No	170/184 (92.4)		173/187 (92.5)				
SI	10/184 (5.4)		9/187 (4.8)				
Severe SI	4/184 (2.2)		5/187 (2.7)				

ES = effect size; logMAR = logarithm of the minimum angle of resolution; SI = sight impaired.

∗ Mean difference for continuous outcomes and risk ratios for dichotomous outcomes.

† At diagnosis, a total of 134 participants showed an intraocular pressure of < 22 mmHg: 62 participants (27.3%) and 72 participants (31.9%) in the trabeculectomy and medicine arms, respectively.

**Figure 4:**
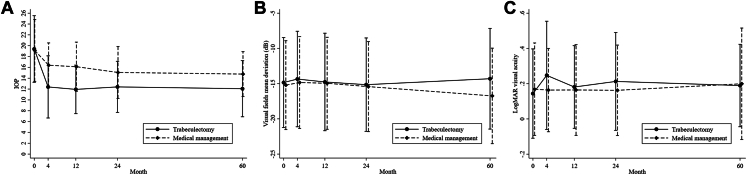
**A**–**C**, Clinical outcomes up to 5-years for (**A**) intraocular pressure (IOP), (**B**) visual field, and (**C**) logarithm of the minimum angle of resolution (logMAR) visual acuity.

A significant and clinically meaningful difference was found in disease (VF) progression between arms at 5 years. The mean ± SD MD was –14.30 ± 7.14 dB for the trabeculectomy arm and –16.74 ± 6.78 dB for the medical management arm, with a mean difference of 1.87 dB (95% CI, 0.87–2.87; *P* < 0.001; Table 4; Fig 4). Sensitivity analyses of multiple imputations were consistent with the main analysis (Table S3).

No difference was found in logarithm of the minimum angle of resolution visual acuity between arms (mean difference, 0.02; 95% CI, –0.04 to 0.08; *P* = 0.48; Table 4; Fig 4). The need for cataract surgery and accordance with visual standards for driving and certification as being sight impaired were similar for both arms (Table 4).

The concentration of mitomycin C used for surgery was left to the discretion of the operating surgeon. Mitomycin C concentration was recorded for 225 of the trabeculectomies undertaken; 74% of cases used the lower 0.2-mg/ml dosage for surgery. In total, 115 participants (52.2%) in the trabeculectomy arm and 124 participants (57.9%) in the medical management arm experienced a safety event during the 5-year follow-up (relative risk, 0.92; 95% CI, 0.72–1.19; *P* = 0.54; Table 5). Twenty participants in both arms experienced a serious AE. Twenty-nine deaths occurred, all unrelated to the trial. Two participants demonstrated endophthalmitis, one in each arm of the study, and 4 participants lost > 10 letters of logarithm of the minimum angle of resolution visual acuity, 3 in the trabeculectomy arm (2 because of progressive glaucoma and 1 because of central serous retinopathy) and 1 in the medical arm (because of progressive glaucoma). No difference was found between groups for these AEs. Hypotony and shallow anterior chamber requiring intervention were not common; the reasons for additional interventions are shown in Table 6. Although in total 249 participants underwent trabeculectomy (173 had split fixation using the definition of the presence at least 1 paracentral quadrant test location with a retinal sensitivity of 0 dB and 78 had VF loss < –22 dB), no episodes of wipeout were reported.

**Table 5:** Safety Events as Allocated Ocular Events for Index Eye

Variable	Trabeculectomy (n = 227)	Medical Management (n = 226)
No. of participants with a safety event, no. (%)	115 (52.2)	124 (57.9)
	Risk ratio, 0.92; 95% confidence interval, 0.72–1.19; *P* = 0.54	

SAE		
No. of participants	20	20
No. of events	22	24
Details		
Death	14	15
Life-threatening	—	1
Hospitalization	3	8
Significant disability	2	—
Important condition	3	2
Expected event		
Yes	3	3
No	18	20
Unknown	1	1
Classification		
General medical (death)	9	15
Unclassified (death)	5	1
General medical	3	7
Related to glaucoma surgery	2	—
General ophthalmology	1	—
Nonglaucoma vision loss	1	1
Glaucoma progression despite treatment	1	—
AE		
No. of participants	106	110
No. of events	206	225
Details		
Drop related	28	97
Ocular surface related	48	55
Nonspecific	35	29
Potential AE related to surgery	13	8
Glaucoma progression	3	12
Hypotony requiring intervention	15	5
Early bleb leak	13	3
Choroidal effusion	9	4
Shallow anterior chamber	8	3
Ptosis	7	3
Irreversible loss of ≥ 10 ETDRS letters	3	1
Corneal epithelial defect	4	—
Hyphema	4	—
Late bleb leak	4	—
Cataract	1	2
Conjunctival buttonhole	3	—
Macular edema	1	1
Suprachoroidal hemorrhage	2	—
Blebitis	2	—
Endophthalmitis		
Endogenous	1	—
Bled related	—	1
Persistent uveitis	1	—
Retinal detachment	—	1
Nonspecific unrelated uveitis	1	—

AE = adverse event; ETDRS = Early Treatment Diabetic Retinopathy Study; SAE = severe adverse event; — = zero.

**Table 6:** Further Surgery

Variable	Trabeculectomy (n = 227)	Medical Management (n = 226)
No. of participants, no. (%)	75 (0.35)	96 (0.46)
	Risk ratio, 0.68; 95% confidence interval, 0.50–0.92; *P* = 0.011	

No. of interventions for those that had at least 1, mean ± SD	1.24 ± 0.46	1.33 ± 0.63
Details of intervention, no.		
Cataract surgery	57	56
Surgery related to previous glaucoma surgery		
Bleb revision	11	2
AC reformation	5	3
Bleb resuturing	6	1
Further glaucoma surgery		
Trabeculectomy	—	48
Deep sclerectomy with spacer	—	1
Deep Sclerectomy	1	—
Late bleb needling ± mitomycin C	3	—
Selective laser trabeculoplasty	3	11
CyPass Micro-Stent (Alcon Laboratories, Inc.)	—	1
iStent inject (Glaukos Corporation)	—	1
Glaucoma drainage device∗	4	1
Supramid removal	1	1
Tube revision	1	—
Cyclodiode laser	2	2

AC = anterior chamber; SD = standard deviation; — = zero.

∗ Tube types: Paul Glaucoma Implant (Advanced Ophthalmic Innovations), n = 2; Ahmed Glaucoma Valve (New World Medical, Inc.), n = 1; Baerveldt Glaucoma Implant (Johnson & Johnson), n = 1; and not stated, n = 1.

The additional glaucoma surgery required in both arms is shown in Table 6. In the medical management arm, 48 participants (21%) required a trabeculectomy for IOP control. The frequency of additional interventions after trabeculectomy (such as bleb revision, anterior chamber reformation, and bleb resuturing) was higher in the trabeculectomy arm but proportionate to the number of trabeculectomies undertaken in each arm. In the trabeculectomy arm, trabeculectomy failure required further surgery in the form of glaucoma drainage devices in 4 participants.

At 5 years, 62 of 175 participants (35.4%) were using IOP-lowering drops in the trabeculectomy arm and 124 of 171 participants (72.5%) were doing so in the medical management arm (RR, 0.48; 95% CI, 0.34–0.67; *P* < 0.001). The mean ± SD number of drops was 0.64 ± 1.01 and 1.54 ± 1.21 in the trabeculectomy and medical management arms, respectively (Table S7, available at www.aaojournal.org).

## Discussion

### Principal Findings

At 5 years, initial surgery was associated with less disease progression compared with medical treatment. No evidence was found of a difference in the primary outcome measure vision-related QoL between treatment arms. This is also true for the general health status and glaucoma QoL measurements undertaken. Trabeculectomy was more effective in lowering IOP. In addition, the trabeculectomy arm required far fewer topical medications for IOP control. Adverse events, including serious AEs, between arms were similar.

### Comparison with Other Studies

A sustained reduction in IOP is recognized to be the most effective method of preventing further VF loss in glaucoma.^5^^,^^26^^,^^27^ A reduction to 12.07 ± 5.18 mmHg in the trabeculectomy arm and 14.8 ± 4.1 mmHg in the medical arm was found at 5 years. This is a clinically important difference and is consistent with clinicians’ interpretation of a clinically important difference between interventions.^28^

The IOP-lowering achieved in the trabeculectomy arm is consistent with current results reported from the National Health Service by Kirwan et al^29^ in a multicenter service evaluation of trabeculectomies and by Stead and King^30^ and Filippopoulos et al,^31^ who specifically reported IOP lowering in eyes with advanced VF loss. It is also consistent with trabeculectomy-related IOP achieved in other recent glaucoma treatment randomized controlled trials undertaken in a variety of health care systems.^32^^,^^33^

The superior efficacy of trabeculectomy is reflected further in the reduced need for topical medications. At 5 years, the number of participants receiving topical medications was 62 of 175 participants (35.4%) in the trabeculectomy arm and 124 of 171 participants (72.5%) in the medical management arm.

Clinicians consider VF to be the most important outcome because this is a measure of functional vision loss^34^ and the main indicator of disease severity. A clinically important and statistically significant difference was found in disease progression, with those in the initial trabeculectomy group having shown almost 2 dB less VF loss compared with those in the initial medication group after 5 years. To our knowledge, the TAGS is the first trial to report a beneficial effect of primary surgery compared with medication regarding disease progression in patients with advanced disease. Reduced VF progression is particularly important in people with severe disease because they have less VF reserve. Differences in disease progression are most likely a consequence of the sustained lower IOP^5^ in the trabeculectomy arm and possibly less reliance on drops, eliminating the potential for poor adherence to contribute to VF progression.^35^ In the TAGS, the mean age of glaucoma diagnosis was 67 years, and the expected mean survival is about 18 years.^36^ A sustained differential in IOP reduction between treatment arms is likely to result with further lifetime divergence in VF preservation.

For patients, the most important outcome of glaucoma management is their ability to continue to live an independent life and maintain their QoL.^37^^,^^38^ For all generic, vision-specific, and glaucoma-specific QoL measures, no statistical difference was found between the interventions at 5 years, suggesting that neither intervention had a greater effect on participants’ QoL, which will help to inform patients when considering treatment options.

Recent publications suggest that measurements of QoL have low power to discriminate between treatment arms in trials of disease-modifying treatments for glaucoma and probably have greater importance in capturing the impact of side effects and adverse effects.^34^^,^^39^ However, glaucoma typically is a slowly progressive disease, and we argue that the lack of evidence of a difference tells us that the difference in QoL between treatments is not large, rather than some degree of failure is characteristic of the tools themselves. With respect to the EQ-5D-5L and Health Utility Index, these are tools of proven value over a range of conditions including numerous eye conditions. More interestingly, the Glaucoma Utility Index is a condition-specific tool, and its scoring was developed using best practice methods and provides utilities specific to this study.^40^ As such, if a substantial difference in QoL were present between groups with respect to glaucoma-specific QoL, then we would have expected to detect this. Having said this, we cannot infer that no difference in QoL exists; that is not what our study was designed to do. Rather, our inference is that changes in clinical measures have not translated into a difference in QoL. It is possible that this may occur in the future, should measures of clinical effect continue to diverge between the randomized arms.

A major concern for clinicians is the perceived high risk of complications associated with trabeculectomy, specifically, the risk of unexplained catastrophic vision loss immediately after surgery (termed "wipeout"), which is believed to be a significant risk in patients with advanced VF loss, and the risk of long-term complications and sight loss associated with trabeculectomy.^9^ At 5 years, no evidence was found to support these concerns. Additional surgery was required in both arms. In the medical management arm, 48 participants (21.4%) had undergone trabeculectomy by 5 years for uncontrolled glaucoma. Additional surgery occurred in both arms to deal with consequences of trabeculectomy, such as hypotony. The rates were proportional between the arms in the context of trabeculectomy surgeries undertaken. However, 8% of the trabeculectomy group did require an intervention to manage clinically significant hypotony, and the need for the possibility of this and other potential additional interventions should be highlighted to patients when considering trabeculectomy as a primary intervention.

### Strengths and Limitations

The TAGS strengths include its pragmatic design to replicate, as closely as possible, current practice and the reality of outcomes in a publicly funded health care system, the large sample size with low attrition rate over a 5-year follow-up, the involvement of multiple centers in the United Kingdom, the randomization process, and the masking of the clinical assessments for IOP and VFs, which kept the potential risk of bias to a minimum.

The surgical treatments could not be masked from participants, nor could some of the clinical outcome assessments such as evaluation of complications. Some of the 5-year data were collected during the restrictions of the coronavirus disease 2019 pandemic, and, consequently, to minimize patient and clinician contact time, the 2-observers technique for IOP measurement was not performed for some IOP measurements, and, for VF assessment, a shorter testing algorithm (24-2 SITA Fast) was used. Some data collection also was incomplete. However, sensitivity analyses demonstrated no evidence that attrition or missing data affected the validity of the results at 60 months. Most participants were White, which reflects the population of the United Kingdom; however, this may limit the generalizability of our findings to non-White populations.

## Conclusions

At 5 years, the TAGS demonstrated that primary trabeculectomy surgery is more effective in lowering IOP and preventing disease progression than primary medical treatment in patients with advanced disease and has a similar safety profile. Trabeculectomy should be offered as a primary intervention in patients with advanced glaucoma.
