# Supplementary material for: Evaluating Primary Treatment for People with Advanced Glaucoma: Five-Year Results of the Treatment of Advanced Glaucoma Study
Source: Ophthalmology. 2024 Jul;131(7):759–70. doi: 10.1016/j.ophtha.2024.01.007 (PMC11190021; doi:10.1016/j.ophtha.2024.01.007)
Supplement: Figure S1 [file mmc1.pdf]

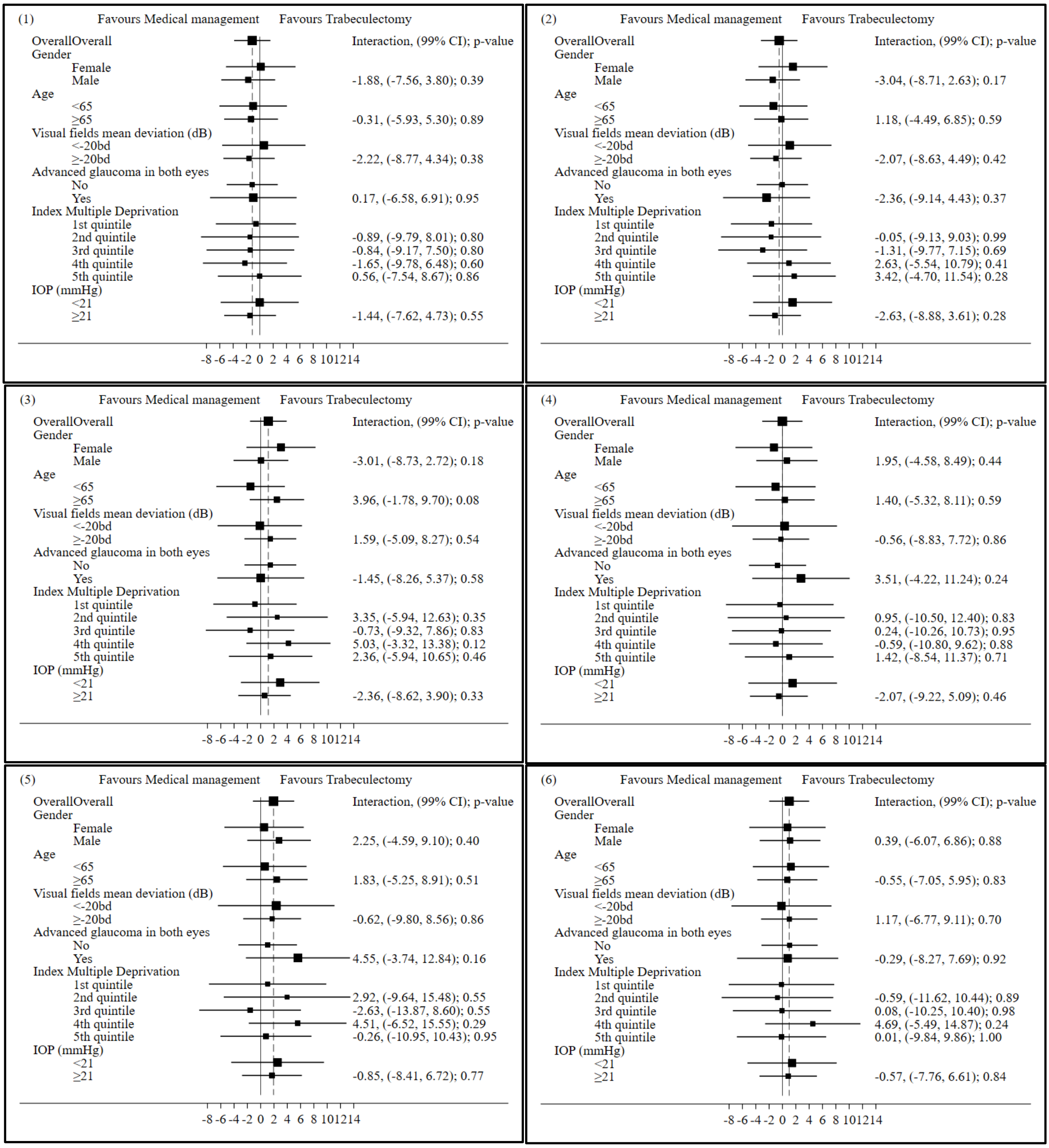

Supplementary Figure 1.

Subgroup analysis of the primary outcome VFQ-25 for trabeculectomy vs medical management: (1) 4 months, (2) 12 months, (3) 24 months, (4) 36 months, (5) 48 months, (6) 60 months.

First quintile, most deprived; fifth quintile, least deprived. Boxes indicate mean differences. Solid line indicates 99% confidence intervals. Solid vertical line indicates no effect. Dashed vertical line indicates overall effect.

Over the different follow-up time points, there are cases where there was a slight change in which subgroup favoured which treatment. For example, Gender, Male favoured medical

management at 4 months but at 60 months it favoured trabeculectomy. However, there was no overall difference.
