# Supplementary material for: Evaluating Primary Treatment for People with Advanced Glaucoma: Five-Year Results of the Treatment of Advanced Glaucoma Study
Source: Ophthalmology. 2024 Jul;131(7):759–70. doi: 10.1016/j.ophtha.2024.01.007 (PMC11190021; doi:10.1016/j.ophtha.2024.01.007)
Supplement: Table S2 [file mmc2.pdf]

VFQ-25 subscales	Trabeculectomy N=227	Medical management N=226	MD	95% CI	p-value
Near activities					
Baseline	84.2 (18.5); 225	84.4 (16.9); 224			
4 months	83.9 (18.0); 211	84.9 (17.1); 214	-0.59	(-4.57 to 3.39)	0.77
12 months	84.1 (18.6); 214	84.7 (18.4); 209	-0.07	(-4.07 to 3.93)	0.97
24 months	82.8 (18.4); 205	82.3 (19.9); 204	1.07	(-2.96 to 5.11)	0.60
36 months	82.4 (19.0); 158	82.2 (19.9); 152	-0.31	(-4.70 to 4.08)	0.89
48 months	83.1 (18.3); 137	80.0 (20.7); 141	2.34	(-2.19 to 6.88)	0.31
60 months	81.5 (20.5); 156	78.5 (20.6); 157	2.10	(-2.27 to 6.47)	0.35
Distance activities					
Baseline	88.5 (16.1); 226	89.7 (14.4); 224			
4 months	87.8 (16.8); 211	89.0 (15.4); 216	-0.12	(-3.42 to 3.19)	0.94
12 months	88.2 (16.3); 214	88.6 (15.7); 209	0.75	(-2.57 to 4.07)	0.66
24 months	88.0 (15.9); 207	86.2 (18.9); 204	2.66	(-0.69 to 6.00)	0.12
36 months	85.7 (18.6); 159	84.8 (18.5); 152	0.52	(-3.15 to 4.19)	0.78
48 months	86.3 (17.5); 138	83.4 (17.9); 141	2.53	(-1.26 to 6.33)	0.19
60 months	84.1 (18.9); 156	82.5 (19.3); 157	1.35	(-2.30 to 5.00)	0.47
Dependency					
Baseline	94.0 (17.3); 226	94.9 (15.7); 222			
4 months	91.2 (20.2); 211	93.4 (17.5); 216	-1.58	(-5.44 to 2.28)	0.42
12 months	92.1 (19.6); 213	94.3 (14.5); 209	-1.26	(-5.14 to 2.62)	0.52
24 months	93.6 (15.6); 206	92.7 (17.7); 203	2.11	(-1.80 to 6.03)	0.29
36 months	92.3 (18.0); 156	91.6 (20.5); 152	1.05	(-3.27 to 5.38)	0.63
48 months	92.8 (16.4); 136	90.8 (20.1); 139	1.44	(-3.05 to 5.93)	0.53
60 months	90.9 (19.3); 154	89.9 (21.4); 157	0.45	(-3.86 to 4.75)	0.84
Driving					
Baseline	85.9 (26.7); 171	84.8 (26.2); 158			
4 months	82.1 (30.6); 151	83.0 (27.4); 155	-0.64	(-8.50 to 7.21)	0.87
12 months	81.3 (28.9); 152	79.9 (29.8); 149	0.07	(-7.86 to 7.99)	0.99
24 months	81.1 (29.3); 143	79.9 (28.7); 138	1.48	(-6.56 to 9.52)	0.72
36 months	78.0 (31.9); 115	68.7 (37.6); 110	6.45	(-2.08 to 14.97)	0.14
48 months	77.9 (30.8); 102	69.6 (36.0); 110	7.04	(-1.58 to 15.67)	0.11
60 months	78.3 (29.8); 108	67.9 (37.2); 120	8.93	(0.45 to 17.41)	0.039
General health					
Baseline	63.6 (23.4); 225	60.9 (22.6); 223			
4 months	66.0 (23.4); 211	64.8 (20.9); 215	-0.43	(-5.22 to 4.37)	0.86
12 months	66.1 (23.8); 214	60.5 (22.4); 208	4.53	(-0.28 to 9.35)	0.07
24 months	63.3 (25.5); 206	59.9 (23.5); 205	2.05	(-2.80 to 6.89)	0.41
36 months	62.1 (23.7); 155	56.3 (22.1); 150	3.52	(-1.77 to 8.81)	0.19
48 months	59.2 (23.9); 136	57.2 (21.3); 139	-0.70	(-6.15 to 4.75)	0.80
60 months	59.3 (25.1); 153	54.5 (21.5); 157	2.01	(-3.25 to 7.26)	0.45
Role difficulties					
Baseline	87.1 (19.8); 226	87.4 (20.8); 222			
4 months	82.5 (24.5); 212	86.3 (21.7); 216	-4.42	(-8.99 to 0.14)	0.06
12 months	83.2 (23.2); 213	84.8 (21.1); 209	-1.87	(-6.46 to 2.72)	0.42

24 months	83.0 (22.7); 207	83.4 (23.4); 203	-0.68	(-5.31 to 3.95)	0.77
36 months	80.2 (26.4); 158	82.6 (24.2); 149	-3.69	(-8.84 to 1.45)	0.16
48 months	81.7 (22.6); 136	80.5 (25.6); 140	-1.25	(-6.60 to 4.10)	0.65
60 months	81.7 (24.5); 154	80.4 (25.2); 157	-0.60	(-5.72 to 4.52)	0.82
Mental health					
Baseline	81.1 (21.2); 226	81.8 (19.9); 224			
4 months	79.5 (23.3); 212	83.5 (19.6); 216	-3.76	(-7.85 to 0.32)	0.07
12 months	79.6 (22.4); 214	83.2 (19.7); 209	-3.23	(-7.34 to 0.87)	0.12
24 months	80.8 (20.7); 207	81.4 (21.6); 205	-0.55	(-4.69 to 3.59)	0.79
36 months	80.7 (20.8); 158	81.5 (22.1); 152	-1.57	(-6.16 to 3.01)	0.50
48 months	81.1 (20.7); 137	79.2 (23.3); 142	0.49	(-4.27 to 5.24)	0.84
60 months	79.4 (21.5); 157	79.3 (23.6); 159	-1.99	(-6.53 to 2.56)	0.39
General vision					
Baseline	74.9 (14.5); 223	72.8 (14.2); 223			
4 months	71.7 (14.4); 211	74.0 (12.5); 215	-3.19	(-6.29 to -0.08)	0.044
12 months	73.1 (14.6); 213	73.8 (13.9); 208	-1.72	(-4.84 to 1.40)	0.28
24 months	73.3 (13.4); 206	72.2 (14.6); 203	-0.06	(-3.21 to 3.09)	0.97
36 months	72.3 (14.6); 158	72.7 (13.9); 151	-1.81	(-5.26 to 1.65)	0.31
48 months	73.4 (13.8); 136	70.2 (15.6); 141	1.75	(-1.83 to 5.33)	0.34
60 months	71.7 (14.4); 155	69.9 (16.0); 159	0.22	(-3.21, to 3.65)	0.90
Social function					
Baseline	95.2 (11.9); 225	94.9 (12.1); 224			
4 months	95.1 (12.0); 211	94.6 (11.9); 216	0.29	(-2.47 to 3.05)	0.84
12 months	94.3 (13.5); 214	94.5 (11.8); 208	-0.34	(-3.12 to 2.44)	0.81
24 months	95.0 (12.2); 206	93.2 (16.1); 205	1.45	(-1.35 to 4.26)	0.31
36 months	93.6 (14.8); 159	92.2 (15.3); 152	1.09	(-2.02 to 4.20)	0.49
48 months	93.4 (14.2); 138	91.5 (15.4); 142	1.51	(-1.71 to 4.73)	0.36
60 months	92.8 (15.0); 157	90.0 (17.0); 159	1.89	(-1.20 to 4.97)	0.23
Colour vision					
Baseline	96.9 (10.9); 223	96.6 (11.1); 222			
4 months	97.4 (8.8); 209	96.8 (10.2); 214	0.38	(-1.90 to 2.65)	0.75
12 months	96.1 (11.1); 212	97.4 (8.7); 205	-1.39	(-3.69 to 0.90)	0.23
24 months	95.6 (13.9); 206	95.0 (15.2); 204	0.67	(-1.63 to 2.98)	0.57
36 months	94.3 (16.1); 159	95.2 (12.9); 150	-0.37	(-3.00 to 2.26)	0.79
48 months	94.6 (13.4); 135	95.5 (13.9); 139	-0.79	(-3.56 to 1.97)	0.57
60 months	94.5 (14.4); 154	93.5 (16.6); 155	0.78	(-1.84 to 3.40)	0.56
Peripheral vision					
Baseline	86.6 (20.8); 224	87.2 (20.2); 224			
4 months	85.4 (21.1); 210	85.6 (20.4); 214	-0.90	(-5.32 to 3.52)	0.69
12 months	86.4 (20.8); 214	85.6 (20.2); 207	0.48	(-3.96 to 4.92)	0.83
24 months	85.1 (19.6); 205	83.8 (22.4); 204	0.44	(-4.03 to 4.92)	0.85
36 months	83.5 (21.2); 159	82.0 (21.4); 151	0.66	(-4.22 to 5.54)	0.79
48 months	82.3 (21.9); 137	79.8 (22.1); 141	1.34	(-3.70 to 6.38)	0.60
60 months	80.4 (22.9); 157	79.6 (23.0); 157	-0.04	(-4.89 to 4.81)	0.99
Ocular pain					
Baseline	84.7 (19.0); 225	83.9 (17.2); 224			
4 months	81.3 (19.2); 212	80.5 (18.9); 216	-0.29	(-4.12 to 3.53)	0.88
12 months	81.6 (20.0); 214	81.9 (16.3); 209	-1.05	(-4.90 to 2.79)	0.59

24 months	81.6 (19.7); 207	80.5 (18.7); 205	0.44	(-3.43 to 4.32)	0.82
36 months	80.8 (20.4); 159	83.1 (16.1); 152	-3.36	(-7.63 to 0.92)	0.12
48 months	82.5 (19.6); 137	81.2 (18.6); 142	0.69	(-3.75 to 5.13)	0.76
60 months	82.1 (18.6); 157	82.2 (17.8); 159	-1.53	(-5.78 to 2.72)	0.48

Supplementary Table 2 - VFQ-25 subscale values
