# Supplementary material for: Evaluating Primary Treatment for People with Advanced Glaucoma: Five-Year Results of the Treatment of Advanced Glaucoma Study
Source: Ophthalmology. 2024 Jul;131(7):759–70. doi: 10.1016/j.ophtha.2024.01.007 (PMC11190021; doi:10.1016/j.ophtha.2024.01.007)
Supplement: Table S3 [file mmc3.pdf]

	MD	95% CI	p-value
**VFQ-25**			
4 months	-0.86	(-4.12, 2.37)	0.60
12 months	-0.07	(-3.33, 3.18)	0.72
24 months	1.49	(-1.76, 4.75)	0.41
36 months	-0.09	(-3.73, 3.56)	0.96
48 months	1.17	(-2.46, 4.81)	0.53
60 months	0.38	(-3.15, 3.92)	0.83
**IOP**			
4 months	-3.97	(-5.11, 2.82)	<0.001
12 months	-4.21	(-5.37, -3.04)	<0.001
24 months	-2.68	(-3.85, -1.50)	<0.001
60 months	-2.75	(-3.99, -1.52)	<0.001
**Visual fields mean deviation (dB)**			
4 months	0.18	(-0.78, 1.14)	0.71
12 months	0.11	(-0.85, 1.08)	0.82
24 months	-0.07	(-1.04, 0.90)	0.89
60 months	1.24	(0.25, 2.22)	0.014

MD mean difference; CI Confidence Interval.

Supplementary Table 3 – multiple imputation for VFQ-25, IOP and Visual fields mean deviation (dB)
