# Supplementary material for: Evaluating Primary Treatment for People with Advanced Glaucoma: Five-Year Results of the Treatment of Advanced Glaucoma Study
Source: Ophthalmology. 2024 Jul;131(7):759–70. doi: 10.1016/j.ophtha.2024.01.007 (PMC11190021; doi:10.1016/j.ophtha.2024.01.007)
Supplement: Table S7 [file mmc4.pdf]

	Trabeculectomy N=227	Medical management N=226
	**N=175**	**N=171**
Number of participants, n (%)	62 (35.4)	124 (72.5)
	RR 0.48	95% CI (0.34 to 0.67); p-value <0.001
Number of glaucoma eye drops for those that received at least one		
mean (SD)	1.81 (0.88)	2.13 (0.88)
Median [25th, 75th Percentile]	2 [1, 2]	2 [1.5, 3]
Number of glaucoma eye drops all participants		
mean (SD)	0.64 (1.01)	1.54 (1.21)
Median [25th, 75th Percentile]	0 [0, 1]	2 [0, 2]

RR incidence risk ratio

Supplementary Table 7 – need for drops at 5-years
